# Methyl 2-(2,2,4-trimethyl-6-tosyl­perhydro-1,3-dioxino[5,4-*c*]pyridin-5-yl)acetate

**DOI:** 10.1107/S1600536809014391

**Published:** 2009-04-22

**Authors:** S. Selvanayagam, B. Sridhar, K. Ravikumar, S. Kathiravan, R. Raghunathan

**Affiliations:** a Department of Physics, Kalasalingam University, Krishnankoil 626 190, India; b Laboratory of X-ray Crystallography, Indian Institute of Chemical Technology, Hyderabad 500 007, India; c Department of Organic Chemistry, University of Madras, Guindy Campus, Chennai 600 025, India

## Abstract

The title compound, C_20_H_29_NO_6_S, crystallizes with two mol­ecules in the asymmetric unit, with similar conformations. The dioxane and pyridine rings adopt twist conformations in both mol­ecules. The packing is stabilized by inter­molecular C—H⋯O hydrogen bonds.

## Related literature

For general background to dioxane derivatives, see: Khali *et al.* (1985 ▶); Li *et al.* (2008 ▶); Sladowska *et al.* (2004 ▶); Schmidt *et al.* (2007 ▶); Tafeenko *et al.* (2008 ▶); Selvanayagam *et al.* (2005 ▶). For puckering data, see: Cremer & Pople (1975 ▶).
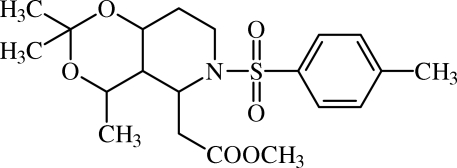


## Experimental

### 

#### Crystal data

C_20_H_29_NO_6_S
*M* *_r_* = 411.50
Orthorhombic, 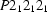

*a* = 8.2379 (16) Å
*b* = 18.039 (4) Å
*c* = 28.844 (6) Å
*V* = 4286.2 (15) Å^3^
*Z* = 8
Mo *K*α radiation
μ = 0.19 mm^−1^
*T* = 293 K
0.25 × 0.23 × 0.21 mm

#### Data collection

Bruker SMART APEX CCD area-detector diffractometer
Absorption correction: none
49982 measured reflections
10142 independent reflections
7073 reflections with *I* > 2σ(*I*)
*R*_int_ = 0.046

#### Refinement

*R*[*F* ^2^ > 2σ(*F* ^2^)] = 0.055
*wR*(*F* ^2^) = 0.124
*S* = 1.04
10142 reflections
515 parameters
H-atom parameters constrained
Δρ_max_ = 0.28 e Å^−3^
Δρ_min_ = −0.16 e Å^−3^
Absolute structure: Flack (1983 ▶), 4401 Friedel pairs
Flack parameter: −0.04 (6)

### 

Data collection: *SMART* (Bruker, 2001 ▶); cell refinement: *SAINT* (Bruker, 2001 ▶); data reduction: *SAINT*; program(s) used to solve structure: *SHELXS97* (Sheldrick, 2008 ▶); program(s) used to refine structure: *SHELXL97* (Sheldrick, 2008 ▶); molecular graphics: *ORTEP-3* (Farrugia, 1997 ▶) and *PLATON* (Spek, 2009 ▶); software used to prepare material for publication: *SHELXL97* and *PARST* (Nardelli, 1995 ▶).

## Supplementary Material

Crystal structure: contains datablocks I, global. DOI: 10.1107/S1600536809014391/bt2928sup1.cif

Structure factors: contains datablocks I. DOI: 10.1107/S1600536809014391/bt2928Isup2.hkl

Additional supplementary materials:  crystallographic information; 3D view; checkCIF report

## Figures and Tables

**Table 1:** Hydrogen-bond geometry (Å, °)

*D*—H⋯*A*	*D*—H	H⋯*A*	*D*⋯*A*	*D*—H⋯*A*
C4*B*—H4*B*2⋯O4*A*^i^	0.97	2.53	3.277 (3)	134
C13*A*—H13*B*⋯O5*B*^ii^	0.96	2.51	3.256 (5)	134
C13*A*—H13*C*⋯O4*B*^iii^	0.96	2.58	3.229 (4)	125
C18*A*—H18*A*⋯O3*A*^iv^	0.93	2.54	3.409 (4)	156
C18*B*—H18*B*⋯O3*B*^v^	0.93	2.56	3.403 (3)	151

Symmetry codes: (i) 
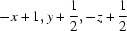
; (ii) 
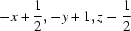
; (iii) 
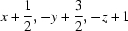
; (iv) 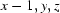; (v) 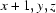.

**Fig. 1.**
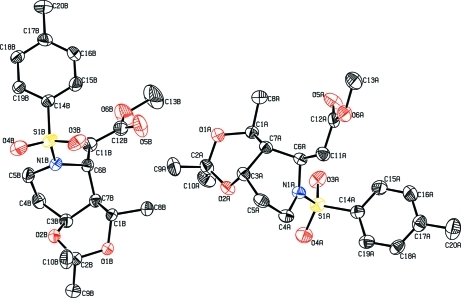
The structure and atom-numbering scheme for (I); displacement ellipsoids are drawn at the 30% probability level and H atoms are omitted for the sake of clarity.

**Fig. 2.**
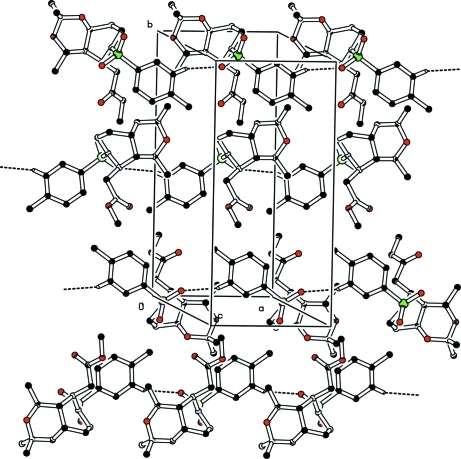
Molecular packing of (I) viewed along the c axis; H-bonds are shown as dashed lines. For the sake of clarity, H atoms, not involved in hydrogen bonds, have been omitted.

## supplementary crystallographic information

### Comment

Dioxane derivatives possess anti-inflammatory (Khali *et al.*, 1985; Li *et al.*, 2008) and pharmacoligical (Sladowska *et al.*, 2004) activities. These derivatives act as effective modulators to overcome multidrug resistance (Schmidt *et al.*, 2007). In view of its importance, we have undertaken the single-crystal X-ray diffraction study and report here its results.

The X-ray study confirmed the molecular structure and atomic connectivity for (I), as illustrated in Fig. 1. The asymmetric unit of (I) contains two molecules (Fig. 1); their corresponding bond lengths and bond angles are in good agreement.     The geometry of the dioxane ring is comparable to the reported literature value (Tafeenko, *et al.*, 2008).

Atom S1 has a distorted tetrahedral configuration in both the molecules with the angles O—S—O and C—S—N (See Table 1) deviating significantly from ideal tetrahedral values. Similar distortions in the sulfonyl group were reported and attributed to the repulsive interaction between short S=O bonds (Selvanayagam *et al.*, 2005). The sums of the angles at atoms N1 of the piperidin ring (358.6 for molecule A and 356.5°, respectively for molecule B) are in accordance with *sp*^3^ hybridization.

The acetate group in both the molecules is planar with a maximum deviation of -0.049 (4) Å for C13 in molecule A and 0.022 (3) Å for C12 in molecule B. The mean planes of the acetate group and tosylmethly groups make a dihedral angle of 56.1 (1)° for molecule A and 49.5 (1)° for molecule B.

The dioxane ring of both the molecule adopts a twist conformation with puckering parameters q_3_ = 0.010 (2), q_2_ = Q_T_ = 0.726 (1), θ = 89.2 (2)° for molecule A and q_3_ = 0.017 (1), q_2_ = Q_T_ = 0.724 (1), θ = 88.6 (2)° for molecule B (Cremer & Pople, 1975). The pyridine ring of both the molecules are also adopt a twist conformation and it is confirmed with puckering parameters.

In addition to van der Waals forces, the molecular packing is stabilized by intra and intermolecular C—H···O hydrogen bonds (Table 2). Atom H18A of C18A in molecule A and H18B of C18B in molecules B forms a intermolecular hydrogen bond with oxygen atom O3 (O3A for molecule A; O3B for molecule B) forming a C(6) chaing motif of C—H···O hydrogen bond in the unit cell (Fig. 2).

### Experimental

The methyl 2-(hexahydro-2,2,4-trimethyl-4*H*-[1,3]dioxino[5,4-*c*]pyridin-5-yl) acetate (0.01 mol) was dissolved in dry DMF and then pottasium carbonate was added and stirred for about 5 to 10 minutes. Then *p*-toluene sulfonyl chloride was added to the solution and extract with ethyl acetate (30 ml). Then the organic solution was dried over sodium sulfate and evaporated under reduced pressure to give the title compound. In order to get the diffraction quality crystals, the compound was recrystallized from hexane and ethyl acetate (1:1) mixture.

### Refinement

The H atoms were positioned geometrically with C—H distances of 0.93–0.98 Å and were included in the refinement in the riding motion approximation with *U*_iso_= 1.5*U*_eq_(C) for methyl H and 1.2*U*_eq_ (C) for other H atoms.

### Crystal data

**Table:**

C_20_H_29_NO_6_S	*F*(000) = 1760
*M**_r_* = 411.50	*D*_x_ = 1.275 Mg m^−^^3^
Orthorhombic, *P*2_1_2_1_2_1_	Mo *K*α radiation, λ = 0.71073 Å
Hall symbol: P 2ac 2ab	Cell parameters from 32218 reflections
*a* = 8.2379 (16) Å	θ = 2.2–25.4°
*b* = 18.039 (4) Å	µ = 0.19 mm^−^^1^
*c* = 28.844 (6) Å	*T* = 293 K
*V* = 4286.2 (15) Å^3^	Block, colourless
*Z* = 8	0.25 × 0.23 × 0.21 mm

### Data collection

**Table:**

Bruker SMART APEX diffractometer	7073 reflections with *I* > 2σ(*I*)
Radiation source: fine-focus sealed tube	*R*_int_ = 0.046
graphite	θ_max_ = 28.0°, θ_min_ = 1.4°
ω scans	*h* = −10→10
49982 measured reflections	*k* = −23→23
10142 independent reflections	*l* = −37→37

### Refinement

**Table:**

Refinement on *F*^2^	Secondary atom site location: difference Fourier map
Least-squares matrix: full	Hydrogen site location: inferred from neighbouring sites
*R*[*F*^2^ > 2σ(*F*^2^)] = 0.055	H-atom parameters constrained
*wR*(*F*^2^) = 0.124	*w* = 1/[σ^2^(*F*_o_^2^) + (0.0589*P*)^2^ + 0.1953*P*] where *P* = (*F*_o_^2^ + 2*F*_c_^2^)/3
*S* = 1.04	(Δ/σ)_max_ = 0.001
10142 reflections	Δρ_max_ = 0.28 e Å^−^^3^
515 parameters	Δρ_min_ = −0.16 e Å^−^^3^
0 restraints	Absolute structure: Flack (1983), 4401 Friedel pairs
Primary atom site location: structure-invariant direct methods	Flack parameter: −0.04 (6)

### Special details

**Table:**

Geometry. All e.s.d.'s (except the e.s.d. in the dihedral angle between two l.s. planes) are estimated using the full covariance matrix. The cell e.s.d.'s are taken into account individually in the estimation of e.s.d.'s in distances, angles and torsion angles; correlations between e.s.d.'s in cell parameters are only used when they are defined by crystal symmetry. An approximate (isotropic) treatment of cell e.s.d.'s is used for estimating e.s.d.'s involving l.s. planes.
Refinement. Refinement of *F*^2^ against ALL reflections. The weighted *R*-factor *wR* and goodness of fit *S* are based on *F*^2^, conventional *R*-factors *R* are based on *F*, with *F* set to zero for negative *F*^2^. The threshold expression of *F*^2^ > σ(*F*^2^) is used only for calculating *R*-factors(gt) *etc*. and is not relevant to the choice of reflections for refinement. *R*-factors based on *F*^2^ are statistically about twice as large as those based on *F*, and *R*- factors based on ALL data will be even larger.

### Fractional atomic coordinates and isotropic or equivalent isotropic displacement parameters (Å^2^)

**Table:**

	*x*	*y*	*z*	*U*_iso_*/*U*_eq_	
S1A	0.57526 (9)	0.51715 (4)	−0.03628 (2)	0.05796 (19)	
O1A	1.0619 (2)	0.59589 (10)	0.06477 (6)	0.0605 (5)	
O2A	0.8212 (2)	0.65856 (9)	0.04766 (6)	0.0603 (5)	
O3A	0.7138 (2)	0.47521 (13)	−0.04998 (6)	0.0755 (6)	
O4A	0.5354 (3)	0.58420 (12)	−0.06000 (7)	0.0772 (6)	
O5A	0.7517 (4)	0.34535 (12)	0.08458 (9)	0.0904 (7)	
O6A	0.5539 (3)	0.35248 (12)	0.13703 (8)	0.0808 (6)	
N1A	0.5972 (3)	0.53785 (11)	0.01758 (7)	0.0513 (5)	
C1A	0.9710 (3)	0.53064 (14)	0.05298 (9)	0.0492 (6)	
H1A	0.9541	0.5292	0.0194	0.059*	
C2A	0.9936 (4)	0.66230 (16)	0.04702 (11)	0.0660 (8)	
C3A	0.7559 (3)	0.61454 (14)	0.08419 (9)	0.0517 (6)	
H3A	0.7974	0.6325	0.1140	0.062*	
C4A	0.5748 (4)	0.62460 (15)	0.08266 (10)	0.0609 (7)	
H4A1	0.5494	0.6766	0.0874	0.073*	
H4A2	0.5260	0.5968	0.1078	0.073*	
C5A	0.5013 (4)	0.59916 (15)	0.03701 (10)	0.0610 (7)	
H5A1	0.3905	0.5829	0.0421	0.073*	
H5A2	0.4994	0.6402	0.0153	0.073*	
C6A	0.6795 (3)	0.48787 (13)	0.05007 (8)	0.0494 (6)	
H6A	0.7388	0.4512	0.0316	0.059*	
C7A	0.8063 (3)	0.53303 (13)	0.07744 (8)	0.0473 (6)	
H7A	0.8186	0.5104	0.1081	0.057*	
C8A	1.0724 (4)	0.46516 (16)	0.06734 (10)	0.0656 (8)	
H8A1	1.1726	0.4654	0.0503	0.098*	
H8A2	1.0143	0.4202	0.0609	0.098*	
H8A3	1.0951	0.4682	0.0999	0.098*	
C9A	1.0572 (5)	0.72345 (17)	0.07810 (13)	0.0942 (11)	
H9A	1.0135	0.7702	0.0682	0.141*	
H9B	1.1735	0.7249	0.0763	0.141*	
H9C	1.0248	0.7140	0.1095	0.141*	
C10A	1.0386 (5)	0.67399 (18)	−0.00378 (11)	0.0861 (11)	
H10A	0.9992	0.6331	−0.0219	0.129*	
H10B	1.1545	0.6771	−0.0067	0.129*	
H10C	0.9903	0.7192	−0.0147	0.129*	
C11A	0.5598 (4)	0.44506 (15)	0.08097 (10)	0.0619 (7)	
H11A	0.5251	0.4767	0.1063	0.074*	
H11B	0.4646	0.4319	0.0630	0.074*	
C12A	0.6349 (4)	0.37600 (16)	0.10034 (11)	0.0637 (8)	
C13A	0.6222 (5)	0.2887 (2)	0.16031 (14)	0.1084 (14)	
H13A	0.6348	0.2489	0.1385	0.163*	
H13B	0.5510	0.2735	0.1849	0.163*	
H13C	0.7262	0.3015	0.1730	0.163*	
C14A	0.4063 (3)	0.45789 (15)	−0.04134 (9)	0.0542 (6)	
C15A	0.4235 (4)	0.38280 (17)	−0.03590 (13)	0.0792 (9)	
H15A	0.5254	0.3621	−0.0310	0.095*	
C16A	0.2869 (5)	0.33837 (18)	−0.03779 (13)	0.0867 (10)	
H16A	0.2985	0.2874	−0.0343	0.104*	
C17A	0.1342 (4)	0.36747 (19)	−0.04477 (11)	0.0707 (8)	
C18A	0.1212 (4)	0.44205 (18)	−0.05118 (9)	0.0646 (8)	
H18A	0.0197	0.4628	−0.0568	0.077*	
C19A	0.2548 (3)	0.48700 (17)	−0.04956 (8)	0.0613 (7)	
H19A	0.2430	0.5378	−0.0541	0.074*	
C20A	−0.0140 (5)	0.3181 (2)	−0.04509 (16)	0.1092 (14)	
H20A	−0.0797	0.3287	−0.0184	0.164*	
H20B	0.0193	0.2671	−0.0443	0.164*	
H20C	−0.0758	0.3271	−0.0727	0.164*	
S1B	0.28509 (8)	0.99757 (4)	0.78584 (2)	0.05622 (18)	
O1B	−0.1954 (2)	1.04905 (9)	0.67240 (6)	0.0605 (5)	
O2B	0.0287 (2)	1.12430 (9)	0.68719 (7)	0.0559 (5)	
O3B	0.1485 (2)	0.95520 (14)	0.80040 (6)	0.0766 (6)	
O4B	0.3229 (3)	1.06604 (12)	0.80809 (7)	0.0753 (6)	
O5B	0.2026 (4)	0.80369 (13)	0.68850 (11)	0.1137 (9)	
O6B	0.4075 (3)	0.80688 (13)	0.63892 (10)	0.1123 (10)	
N1B	0.2609 (2)	1.01569 (11)	0.73141 (7)	0.0493 (5)	
C1B	−0.0958 (3)	0.99191 (13)	0.69158 (8)	0.0472 (5)	
H1B	−0.0836	1.0000	0.7250	0.057*	
C2B	−0.1430 (4)	1.12199 (16)	0.68368 (12)	0.0676 (8)	
C3B	0.1124 (3)	1.07542 (14)	0.65631 (9)	0.0542 (7)	
H3B	0.0795	1.0859	0.6243	0.065*	
C4B	0.2911 (4)	1.09264 (16)	0.66211 (9)	0.0609 (7)	
H4B1	0.3099	1.1443	0.6546	0.073*	
H4B2	0.3532	1.0628	0.6405	0.073*	
C5B	0.3509 (3)	1.07768 (15)	0.71098 (10)	0.0603 (7)	
H5B1	0.4658	1.0660	0.7103	0.072*	
H5B2	0.3362	1.1217	0.7299	0.072*	
C6B	0.1994 (3)	0.95835 (14)	0.69959 (8)	0.0470 (6)	
H6B	0.1432	0.9211	0.7184	0.056*	
C7B	0.0722 (3)	0.99495 (13)	0.66821 (8)	0.0473 (6)	
H7B	0.0666	0.9667	0.6392	0.057*	
C8B	−0.1829 (4)	0.91985 (14)	0.68341 (10)	0.0584 (7)	
H8B1	−0.2839	0.9200	0.7000	0.088*	
H8B2	−0.1167	0.8795	0.6941	0.088*	
H8B3	−0.2038	0.9140	0.6509	0.088*	
C9B	−0.2052 (5)	1.17063 (18)	0.64537 (15)	0.1038 (13)	
H9D	−0.1790	1.2214	0.6520	0.156*	
H9E	−0.3208	1.1653	0.6429	0.156*	
H9F	−0.1555	1.1563	0.6166	0.156*	
C10B	−0.2031 (4)	1.14526 (18)	0.73155 (14)	0.0929 (11)	
H10D	−0.1633	1.1111	0.7544	0.139*	
H10E	−0.3196	1.1452	0.7319	0.139*	
H10F	−0.1641	1.1942	0.7385	0.139*	
C11B	0.3353 (3)	0.91821 (15)	0.67257 (10)	0.0608 (7)	
H11C	0.3454	0.9403	0.6421	0.073*	
H11D	0.4375	0.9252	0.6887	0.073*	
C12B	0.3041 (4)	0.83826 (17)	0.66744 (12)	0.0697 (8)	
C13B	0.3901 (7)	0.7273 (2)	0.6319 (2)	0.159 (2)	
H13D	0.4124	0.7018	0.6604	0.239*	
H13E	0.4652	0.7111	0.6085	0.239*	
H13F	0.2813	0.7164	0.6221	0.239*	
C14B	0.4565 (3)	0.93962 (14)	0.79184 (8)	0.0475 (6)	
C15B	0.4427 (4)	0.86421 (16)	0.78539 (10)	0.0650 (7)	
H15B	0.3421	0.8430	0.7792	0.078*	
C16B	0.5791 (4)	0.82082 (15)	0.78818 (11)	0.0712 (8)	
H16B	0.5698	0.7700	0.7835	0.085*	
C17B	0.7301 (4)	0.85065 (16)	0.79777 (9)	0.0604 (7)	
C18B	0.7406 (3)	0.92607 (15)	0.80480 (9)	0.0562 (7)	
H18B	0.8405	0.9472	0.8119	0.067*	
C19B	0.6060 (3)	0.97025 (15)	0.80147 (8)	0.0509 (6)	
H19B	0.6155	1.0212	0.8057	0.061*	
C20B	0.8779 (4)	0.80270 (19)	0.80020 (13)	0.0909 (11)	
H20D	0.9688	0.8287	0.7872	0.136*	
H20E	0.8594	0.7579	0.7830	0.136*	
H20F	0.9005	0.7906	0.8320	0.136*	

### Atomic displacement parameters (Å^2^)

**Table:**

	*U*^11^	*U*^22^	*U*^33^	*U*^12^	*U*^13^	*U*^23^
S1A	0.0567 (4)	0.0708 (4)	0.0463 (3)	0.0023 (4)	0.0035 (3)	0.0015 (3)
O1A	0.0601 (11)	0.0523 (11)	0.0689 (12)	−0.0043 (9)	−0.0045 (10)	0.0044 (9)
O2A	0.0675 (13)	0.0481 (10)	0.0652 (12)	0.0024 (9)	0.0035 (10)	0.0073 (9)
O3A	0.0577 (11)	0.1072 (16)	0.0617 (12)	0.0048 (12)	0.0064 (10)	−0.0170 (11)
O4A	0.0927 (16)	0.0832 (14)	0.0558 (11)	−0.0063 (12)	−0.0030 (11)	0.0234 (10)
O5A	0.108 (2)	0.0564 (12)	0.1065 (18)	0.0171 (14)	0.0188 (16)	0.0143 (12)
O6A	0.0797 (15)	0.0733 (14)	0.0895 (15)	−0.0257 (12)	−0.0035 (12)	0.0317 (12)
N1A	0.0557 (13)	0.0497 (12)	0.0485 (12)	0.0049 (11)	−0.0003 (10)	0.0028 (9)
C1A	0.0530 (15)	0.0499 (15)	0.0447 (13)	0.0022 (12)	0.0001 (11)	0.0018 (11)
C2A	0.071 (2)	0.0507 (16)	0.076 (2)	−0.0063 (15)	0.0079 (16)	0.0053 (15)
C3A	0.0650 (18)	0.0475 (14)	0.0425 (13)	0.0003 (13)	0.0026 (12)	−0.0030 (11)
C4A	0.0711 (19)	0.0526 (15)	0.0589 (16)	0.0139 (15)	0.0118 (15)	−0.0032 (13)
C5A	0.0601 (17)	0.0570 (16)	0.0657 (18)	0.0118 (14)	0.0008 (14)	0.0016 (15)
C6A	0.0566 (14)	0.0430 (13)	0.0487 (13)	0.0049 (12)	0.0015 (11)	0.0038 (11)
C7A	0.0587 (15)	0.0446 (13)	0.0386 (12)	0.0020 (12)	0.0006 (11)	0.0044 (10)
C8A	0.0645 (18)	0.0677 (18)	0.0645 (17)	0.0092 (16)	−0.0028 (15)	0.0049 (14)
C9A	0.099 (3)	0.0596 (19)	0.123 (3)	−0.0177 (19)	−0.007 (2)	−0.0115 (19)
C10A	0.101 (3)	0.074 (2)	0.084 (2)	0.0012 (19)	0.022 (2)	0.0239 (18)
C11A	0.0567 (17)	0.0545 (16)	0.0744 (19)	−0.0021 (14)	0.0061 (15)	0.0083 (14)
C12A	0.069 (2)	0.0474 (16)	0.074 (2)	−0.0174 (16)	−0.0092 (16)	0.0045 (15)
C13A	0.122 (3)	0.082 (2)	0.122 (3)	−0.030 (2)	−0.017 (3)	0.053 (2)
C14A	0.0586 (16)	0.0574 (16)	0.0466 (14)	0.0079 (14)	−0.0007 (12)	−0.0067 (12)
C15A	0.0616 (19)	0.068 (2)	0.108 (3)	0.0146 (17)	−0.0082 (19)	−0.0155 (19)
C16A	0.083 (2)	0.0564 (18)	0.120 (3)	0.0020 (19)	−0.008 (2)	−0.0133 (19)
C17A	0.068 (2)	0.078 (2)	0.0661 (19)	−0.0025 (17)	0.0005 (16)	−0.0210 (16)
C18A	0.0550 (17)	0.084 (2)	0.0549 (17)	0.0062 (16)	−0.0058 (13)	−0.0155 (15)
C19A	0.0692 (18)	0.0671 (18)	0.0476 (14)	0.0130 (16)	−0.0053 (13)	−0.0058 (13)
C20A	0.088 (3)	0.110 (3)	0.130 (3)	−0.025 (2)	0.002 (3)	−0.026 (3)
S1B	0.0426 (3)	0.0804 (5)	0.0457 (3)	0.0074 (4)	0.0006 (3)	−0.0030 (3)
O1B	0.0510 (10)	0.0468 (10)	0.0838 (13)	−0.0016 (9)	−0.0206 (10)	−0.0018 (9)
O2B	0.0494 (10)	0.0466 (10)	0.0717 (12)	−0.0047 (8)	−0.0057 (9)	−0.0033 (9)
O3B	0.0471 (11)	0.1263 (18)	0.0563 (12)	−0.0014 (12)	0.0048 (9)	0.0147 (12)
O4B	0.0738 (14)	0.0897 (14)	0.0624 (12)	0.0262 (12)	−0.0113 (10)	−0.0296 (11)
O5B	0.0986 (19)	0.0698 (15)	0.173 (3)	−0.0186 (15)	0.036 (2)	−0.0314 (16)
O6B	0.107 (2)	0.0767 (16)	0.153 (2)	0.0158 (16)	0.0512 (19)	−0.0334 (15)
N1B	0.0429 (11)	0.0553 (12)	0.0498 (11)	−0.0027 (10)	−0.0015 (9)	−0.0023 (10)
C1B	0.0437 (12)	0.0466 (13)	0.0514 (13)	−0.0017 (12)	−0.0082 (10)	−0.0041 (11)
C2B	0.0532 (17)	0.0511 (17)	0.099 (2)	−0.0005 (14)	−0.0173 (16)	−0.0008 (16)
C3B	0.0640 (17)	0.0548 (16)	0.0439 (14)	−0.0072 (14)	−0.0013 (12)	0.0067 (12)
C4B	0.0617 (17)	0.0583 (16)	0.0627 (17)	−0.0125 (15)	0.0156 (15)	0.0070 (13)
C5B	0.0510 (15)	0.0593 (16)	0.0705 (18)	−0.0125 (13)	0.0001 (14)	−0.0032 (14)
C6B	0.0420 (13)	0.0500 (14)	0.0489 (14)	−0.0026 (11)	0.0035 (11)	−0.0002 (11)
C7B	0.0527 (14)	0.0490 (14)	0.0402 (12)	−0.0046 (13)	−0.0023 (10)	−0.0036 (11)
C8B	0.0540 (16)	0.0551 (16)	0.0662 (17)	−0.0091 (13)	−0.0019 (14)	0.0011 (13)
C9B	0.091 (3)	0.064 (2)	0.156 (4)	−0.002 (2)	−0.050 (3)	0.019 (2)
C10B	0.0585 (18)	0.075 (2)	0.145 (3)	−0.0070 (17)	0.009 (2)	−0.043 (2)
C11B	0.0516 (16)	0.0651 (19)	0.0657 (18)	0.0031 (13)	0.0088 (14)	−0.0024 (14)
C12B	0.0538 (18)	0.0617 (19)	0.094 (2)	0.0070 (16)	0.0001 (17)	−0.0127 (17)
C13B	0.151 (5)	0.079 (3)	0.247 (6)	0.022 (3)	0.043 (4)	−0.065 (3)
C14B	0.0446 (14)	0.0578 (16)	0.0402 (13)	0.0002 (12)	0.0002 (11)	0.0031 (12)
C15B	0.0558 (16)	0.0672 (19)	0.0721 (19)	−0.0124 (15)	−0.0072 (15)	0.0101 (15)
C16B	0.082 (2)	0.0458 (16)	0.086 (2)	−0.0046 (16)	−0.0109 (19)	0.0071 (15)
C17B	0.0641 (18)	0.0584 (17)	0.0586 (17)	0.0060 (15)	−0.0053 (14)	0.0101 (13)
C18B	0.0444 (15)	0.0662 (18)	0.0580 (16)	−0.0008 (13)	−0.0039 (12)	0.0064 (13)
C19B	0.0473 (14)	0.0533 (15)	0.0521 (15)	0.0002 (13)	−0.0023 (11)	−0.0012 (12)
C20B	0.082 (2)	0.082 (2)	0.109 (3)	0.026 (2)	−0.013 (2)	0.009 (2)

### Geometric parameters (Å, °)

**Table:**

S1A—O3A	1.425 (2)	S1B—O3B	1.424 (2)
S1A—O4A	1.428 (2)	S1B—O4B	1.426 (2)
S1A—N1A	1.608 (2)	S1B—N1B	1.616 (2)
S1A—C14A	1.761 (3)	S1B—C14B	1.766 (3)
O1A—C2A	1.419 (3)	O1B—C2B	1.422 (3)
O1A—C1A	1.436 (3)	O1B—C1B	1.429 (3)
O2A—C2A	1.421 (4)	O2B—C2B	1.419 (3)
O2A—C3A	1.425 (3)	O2B—C3B	1.431 (3)
O5A—C12A	1.199 (4)	O5B—C12B	1.207 (4)
O6A—C12A	1.321 (4)	O6B—C12B	1.313 (4)
O6A—C13A	1.446 (4)	O6B—C13B	1.457 (4)
N1A—C6A	1.466 (3)	N1B—C5B	1.465 (3)
N1A—C5A	1.470 (3)	N1B—C6B	1.473 (3)
C1A—C8A	1.505 (4)	C1B—C8B	1.503 (3)
C1A—C7A	1.530 (4)	C1B—C7B	1.540 (3)
C1A—H1A	0.9800	C1B—H1B	0.9800
C2A—C9A	1.515 (4)	C2B—C9B	1.501 (4)
C2A—C10A	1.526 (4)	C2B—C10B	1.526 (5)
C3A—C4A	1.504 (4)	C3B—C4B	1.514 (4)
C3A—C7A	1.540 (3)	C3B—C7B	1.528 (3)
C3A—H3A	0.9800	C3B—H3B	0.9800
C4A—C5A	1.520 (4)	C4B—C5B	1.517 (4)
C4A—H4A1	0.9700	C4B—H4B1	0.9700
C4A—H4A2	0.9700	C4B—H4B2	0.9700
C5A—H5A1	0.9700	C5B—H5B1	0.9700
C5A—H5A2	0.9700	C5B—H5B2	0.9700
C6A—C11A	1.537 (4)	C6B—C7B	1.534 (3)
C6A—C7A	1.542 (3)	C6B—C11B	1.544 (4)
C6A—H6A	0.9800	C6B—H6B	0.9800
C7A—H7A	0.9800	C7B—H7B	0.9800
C8A—H8A1	0.9600	C8B—H8B1	0.9600
C8A—H8A2	0.9600	C8B—H8B2	0.9600
C8A—H8A3	0.9600	C8B—H8B3	0.9600
C9A—H9A	0.9600	C9B—H9D	0.9600
C9A—H9B	0.9600	C9B—H9E	0.9600
C9A—H9C	0.9600	C9B—H9F	0.9600
C10A—H10A	0.9600	C10B—H10D	0.9600
C10A—H10B	0.9600	C10B—H10E	0.9600
C10A—H10C	0.9600	C10B—H10F	0.9600
C11A—C12A	1.499 (4)	C11B—C12B	1.472 (4)
C11A—H11A	0.9700	C11B—H11C	0.9700
C11A—H11B	0.9700	C11B—H11D	0.9700
C13A—H13A	0.9600	C13B—H13D	0.9600
C13A—H13B	0.9600	C13B—H13E	0.9600
C13A—H13C	0.9600	C13B—H13F	0.9600
C14A—C15A	1.371 (4)	C14B—C15B	1.378 (4)
C14A—C19A	1.375 (4)	C14B—C19B	1.378 (3)
C15A—C16A	1.382 (5)	C15B—C16B	1.372 (4)
C15A—H15A	0.9300	C15B—H15B	0.9300
C16A—C17A	1.378 (5)	C16B—C17B	1.383 (4)
C16A—H16A	0.9300	C16B—H16B	0.9300
C17A—C18A	1.362 (4)	C17B—C18B	1.378 (4)
C17A—C20A	1.512 (5)	C17B—C20B	1.495 (4)
C18A—C19A	1.368 (4)	C18B—C19B	1.369 (4)
C18A—H18A	0.9300	C18B—H18B	0.9300
C19A—H19A	0.9300	C19B—H19B	0.9300
C20A—H20A	0.9600	C20B—H20D	0.9600
C20A—H20B	0.9600	C20B—H20E	0.9600
C20A—H20C	0.9600	C20B—H20F	0.9600
			
O3A—S1A—O4A	120.1 (1)	O3B—S1B—O4B	120.3 (1)
O3A—S1A—N1A	107.52 (11)	O3B—S1B—N1B	107.33 (11)
O4A—S1A—N1A	106.98 (12)	O4B—S1B—N1B	106.79 (12)
O3A—S1A—C14A	106.73 (12)	O3B—S1B—C14B	106.59 (12)
O4A—S1A—C14A	107.02 (13)	O4B—S1B—C14B	107.10 (12)
N1A—S1A—C14A	108.1 (1)	N1B—S1B—C14B	108.3 (1)
C2A—O1A—C1A	113.6 (2)	C2B—O1B—C1B	113.88 (19)
C2A—O2A—C3A	114.4 (2)	C2B—O2B—C3B	114.7 (2)
C12A—O6A—C13A	115.5 (3)	C12B—O6B—C13B	116.6 (3)
C6A—N1A—C5A	117.9 (2)	C5B—N1B—C6B	117.3 (2)
C6A—N1A—S1A	121.8 (2)	C5B—N1B—S1B	118.9 (2)
C5A—N1A—S1A	118.9 (2)	C6B—N1B—S1B	120.4 (2)
O1A—C1A—C8A	106.8 (2)	O1B—C1B—C8B	106.80 (19)
O1A—C1A—C7A	109.3 (2)	O1B—C1B—C7B	108.72 (19)
C8A—C1A—C7A	112.8 (2)	C8B—C1B—C7B	113.0 (2)
O1A—C1A—H1A	109.3	O1B—C1B—H1B	109.4
C8A—C1A—H1A	109.3	C8B—C1B—H1B	109.4
C7A—C1A—H1A	109.3	C7B—C1B—H1B	109.4
O1A—C2A—O2A	110.6 (2)	O2B—C2B—O1B	110.2 (2)
O1A—C2A—C9A	105.3 (3)	O2B—C2B—C9B	112.0 (3)
O2A—C2A—C9A	111.9 (3)	O1B—C2B—C9B	105.6 (2)
O1A—C2A—C10A	111.5 (2)	O2B—C2B—C10B	104.5 (3)
O2A—C2A—C10A	105.2 (3)	O1B—C2B—C10B	111.3 (3)
C9A—C2A—C10A	112.5 (3)	C9B—C2B—C10B	113.3 (3)
O2A—C3A—C4A	106.6 (2)	O2B—C3B—C4B	105.9 (2)
O2A—C3A—C7A	109.7 (2)	O2B—C3B—C7B	109.9 (2)
C4A—C3A—C7A	112.3 (2)	C4B—C3B—C7B	112.4 (2)
O2A—C3A—H3A	109.4	O2B—C3B—H3B	109.5
C4A—C3A—H3A	109.4	C4B—C3B—H3B	109.5
C7A—C3A—H3A	109.4	C7B—C3B—H3B	109.5
C3A—C4A—C5A	112.6 (2)	C3B—C4B—C5B	112.4 (2)
C3A—C4A—H4A1	109.1	C3B—C4B—H4B1	109.1
C5A—C4A—H4A1	109.1	C5B—C4B—H4B1	109.1
C3A—C4A—H4A2	109.1	C3B—C4B—H4B2	109.1
C5A—C4A—H4A2	109.1	C5B—C4B—H4B2	109.1
H4A1—C4A—H4A2	107.8	H4B1—C4B—H4B2	107.8
N1A—C5A—C4A	110.1 (2)	N1B—C5B—C4B	110.2 (2)
N1A—C5A—H5A1	109.6	N1B—C5B—H5B1	109.6
C4A—C5A—H5A1	109.6	C4B—C5B—H5B1	109.6
N1A—C5A—H5A2	109.6	N1B—C5B—H5B2	109.6
C4A—C5A—H5A2	109.6	C4B—C5B—H5B2	109.6
H5A1—C5A—H5A2	108.2	H5B1—C5B—H5B2	108.1
N1A—C6A—C11A	112.5 (2)	N1B—C6B—C7B	107.50 (19)
N1A—C6A—C7A	108.38 (19)	N1B—C6B—C11B	113.2 (2)
C11A—C6A—C7A	113.8 (2)	C7B—C6B—C11B	113.53 (19)
N1A—C6A—H6A	107.3	N1B—C6B—H6B	107.4
C11A—C6A—H6A	107.3	C7B—C6B—H6B	107.4
C7A—C6A—H6A	107.3	C11B—C6B—H6B	107.4
C1A—C7A—C3A	108.9 (2)	C3B—C7B—C6B	113.2 (2)
C1A—C7A—C6A	110.47 (19)	C3B—C7B—C1B	109.1 (2)
C3A—C7A—C6A	112.7 (2)	C6B—C7B—C1B	109.90 (18)
C1A—C7A—H7A	108.2	C3B—C7B—H7B	108.2
C3A—C7A—H7A	108.2	C6B—C7B—H7B	108.2
C6A—C7A—H7A	108.2	C1B—C7B—H7B	108.2
C1A—C8A—H8A1	109.5	C1B—C8B—H8B1	109.5
C1A—C8A—H8A2	109.5	C1B—C8B—H8B2	109.5
H8A1—C8A—H8A2	109.5	H8B1—C8B—H8B2	109.5
C1A—C8A—H8A3	109.5	C1B—C8B—H8B3	109.5
H8A1—C8A—H8A3	109.5	H8B1—C8B—H8B3	109.5
H8A2—C8A—H8A3	109.5	H8B2—C8B—H8B3	109.5
C2A—C9A—H9A	109.5	C2B—C9B—H9D	109.5
C2A—C9A—H9B	109.5	C2B—C9B—H9E	109.5
H9A—C9A—H9B	109.5	H9D—C9B—H9E	109.5
C2A—C9A—H9C	109.5	C2B—C9B—H9F	109.5
H9A—C9A—H9C	109.5	H9D—C9B—H9F	109.5
H9B—C9A—H9C	109.5	H9E—C9B—H9F	109.5
C2A—C10A—H10A	109.5	C2B—C10B—H10D	109.5
C2A—C10A—H10B	109.5	C2B—C10B—H10E	109.5
H10A—C10A—H10B	109.5	H10D—C10B—H10E	109.5
C2A—C10A—H10C	109.5	C2B—C10B—H10F	109.5
H10A—C10A—H10C	109.5	H10D—C10B—H10F	109.5
H10B—C10A—H10C	109.5	H10E—C10B—H10F	109.5
C12A—C11A—C6A	111.7 (2)	C12B—C11B—C6B	112.5 (2)
C12A—C11A—H11A	109.3	C12B—C11B—H11C	109.1
C6A—C11A—H11A	109.3	C6B—C11B—H11C	109.1
C12A—C11A—H11B	109.3	C12B—C11B—H11D	109.1
C6A—C11A—H11B	109.3	C6B—C11B—H11D	109.1
H11A—C11A—H11B	107.9	H11C—C11B—H11D	107.8
O5A—C12A—O6A	124.1 (3)	O5B—C12B—O6B	122.8 (3)
O5A—C12A—C11A	125.0 (3)	O5B—C12B—C11B	125.2 (3)
O6A—C12A—C11A	110.9 (3)	O6B—C12B—C11B	111.8 (3)
O6A—C13A—H13A	109.5	O6B—C13B—H13D	109.5
O6A—C13A—H13B	109.5	O6B—C13B—H13E	109.5
H13A—C13A—H13B	109.5	H13D—C13B—H13E	109.5
O6A—C13A—H13C	109.5	O6B—C13B—H13F	109.5
H13A—C13A—H13C	109.5	H13D—C13B—H13F	109.5
H13B—C13A—H13C	109.5	H13E—C13B—H13F	109.5
C15A—C14A—C19A	119.4 (3)	C15B—C14B—C19B	119.8 (3)
C15A—C14A—S1A	120.6 (2)	C15B—C14B—S1B	120.4 (2)
C19A—C14A—S1A	120.0 (2)	C19B—C14B—S1B	119.8 (2)
C14A—C15A—C16A	119.0 (3)	C16B—C15B—C14B	119.2 (3)
C14A—C15A—H15A	120.5	C16B—C15B—H15B	120.4
C16A—C15A—H15A	120.5	C14B—C15B—H15B	120.4
C17A—C16A—C15A	121.9 (3)	C15B—C16B—C17B	121.8 (3)
C17A—C16A—H16A	119.1	C15B—C16B—H16B	119.1
C15A—C16A—H16A	119.1	C17B—C16B—H16B	119.1
C18A—C17A—C16A	117.9 (3)	C18B—C17B—C16B	118.0 (3)
C18A—C17A—C20A	121.2 (3)	C18B—C17B—C20B	120.9 (3)
C16A—C17A—C20A	120.9 (3)	C16B—C17B—C20B	121.1 (3)
C17A—C18A—C19A	121.2 (3)	C19B—C18B—C17B	120.9 (3)
C17A—C18A—H18A	119.4	C19B—C18B—H18B	119.5
C19A—C18A—H18A	119.4	C17B—C18B—H18B	119.5
C18A—C19A—C14A	120.7 (3)	C18B—C19B—C14B	120.3 (2)
C18A—C19A—H19A	119.7	C18B—C19B—H19B	119.9
C14A—C19A—H19A	119.7	C14B—C19B—H19B	119.9
C17A—C20A—H20A	109.5	C17B—C20B—H20D	109.5
C17A—C20A—H20B	109.5	C17B—C20B—H20E	109.5
H20A—C20A—H20B	109.5	H20D—C20B—H20E	109.5
C17A—C20A—H20C	109.5	C17B—C20B—H20F	109.5
H20A—C20A—H20C	109.5	H20D—C20B—H20F	109.5
H20B—C20A—H20C	109.5	H20E—C20B—H20F	109.5
			
O3A—S1A—N1A—C6A	−33.3 (2)	O3B—S1B—N1B—C5B	159.7 (2)
O4A—S1A—N1A—C6A	−163.5 (2)	O4B—S1B—N1B—C5B	29.5 (2)
C14A—S1A—N1A—C6A	81.6 (2)	C14B—S1B—N1B—C5B	−85.5 (2)
O3A—S1A—N1A—C5A	161.1 (2)	O3B—S1B—N1B—C6B	−41.6 (2)
O4A—S1A—N1A—C5A	30.8 (2)	O4B—S1B—N1B—C6B	−171.85 (18)
C14A—S1A—N1A—C5A	−84.1 (2)	C14B—S1B—N1B—C6B	73.12 (19)
C2A—O1A—C1A—C8A	170.9 (2)	C2B—O1B—C1B—C8B	170.5 (2)
C2A—O1A—C1A—C7A	−66.8 (3)	C2B—O1B—C1B—C7B	−67.2 (3)
C1A—O1A—C2A—O2A	35.7 (3)	C3B—O2B—C2B—O1B	33.5 (3)
C1A—O1A—C2A—C9A	156.7 (2)	C3B—O2B—C2B—C9B	−83.8 (3)
C1A—O1A—C2A—C10A	−80.9 (3)	C3B—O2B—C2B—C10B	153.2 (2)
C3A—O2A—C2A—O1A	30.8 (3)	C1B—O1B—C2B—O2B	33.1 (3)
C3A—O2A—C2A—C9A	−86.3 (3)	C1B—O1B—C2B—C9B	154.3 (3)
C3A—O2A—C2A—C10A	151.3 (2)	C1B—O1B—C2B—C10B	−82.4 (3)
C2A—O2A—C3A—C4A	173.4 (2)	C2B—O2B—C3B—C4B	174.1 (2)
C2A—O2A—C3A—C7A	−64.8 (3)	C2B—O2B—C3B—C7B	−64.3 (3)
O2A—C3A—C4A—C5A	60.8 (3)	O2B—C3B—C4B—C5B	62.0 (3)
C7A—C3A—C4A—C5A	−59.3 (3)	C7B—C3B—C4B—C5B	−58.0 (3)
C6A—N1A—C5A—C4A	30.9 (3)	C6B—N1B—C5B—C4B	29.6 (3)
S1A—N1A—C5A—C4A	−162.8 (2)	S1B—N1B—C5B—C4B	−171.03 (19)
C3A—C4A—C5A—N1A	30.8 (3)	C3B—C4B—C5B—N1B	32.0 (3)
C5A—N1A—C6A—C11A	63.3 (3)	C5B—N1B—C6B—C7B	−65.0 (3)
S1A—N1A—C6A—C11A	−102.5 (2)	S1B—N1B—C6B—C7B	136.05 (17)
C5A—N1A—C6A—C7A	−63.4 (3)	C5B—N1B—C6B—C11B	61.2 (3)
S1A—N1A—C6A—C7A	130.82 (19)	S1B—N1B—C6B—C11B	−97.7 (2)
O1A—C1A—C7A—C3A	29.2 (3)	O2B—C3B—C7B—C6B	−96.2 (2)
C8A—C1A—C7A—C3A	147.9 (2)	C4B—C3B—C7B—C6B	21.5 (3)
O1A—C1A—C7A—C6A	153.59 (19)	O2B—C3B—C7B—C1B	26.5 (3)
C8A—C1A—C7A—C6A	−87.8 (2)	C4B—C3B—C7B—C1B	144.1 (2)
O2A—C3A—C7A—C1A	30.3 (3)	N1B—C6B—C7B—C3B	34.8 (3)
C4A—C3A—C7A—C1A	148.6 (2)	C11B—C6B—C7B—C3B	−91.2 (3)
O2A—C3A—C7A—C6A	−92.7 (2)	N1B—C6B—C7B—C1B	−87.4 (2)
C4A—C3A—C7A—C6A	25.6 (3)	C11B—C6B—C7B—C1B	146.6 (2)
N1A—C6A—C7A—C1A	−91.5 (2)	O1B—C1B—C7B—C3B	32.7 (2)
C11A—C6A—C7A—C1A	142.6 (2)	C8B—C1B—C7B—C3B	151.1 (2)
N1A—C6A—C7A—C3A	30.7 (3)	O1B—C1B—C7B—C6B	157.36 (19)
C11A—C6A—C7A—C3A	−95.3 (2)	C8B—C1B—C7B—C6B	−84.2 (2)
N1A—C6A—C11A—C12A	158.5 (2)	N1B—C6B—C11B—C12B	141.0 (2)
C7A—C6A—C11A—C12A	−77.8 (3)	C7B—C6B—C11B—C12B	−96.1 (3)
C13A—O6A—C12A—O5A	4.6 (4)	C13B—O6B—C12B—O5B	2.7 (6)
C13A—O6A—C12A—C11A	−176.2 (3)	C13B—O6B—C12B—C11B	178.8 (4)
C6A—C11A—C12A—O5A	−20.3 (4)	C6B—C11B—C12B—O5B	−13.2 (5)
C6A—C11A—C12A—O6A	160.4 (2)	C6B—C11B—C12B—O6B	170.8 (3)
O3A—S1A—C14A—C15A	28.9 (3)	O3B—S1B—C14B—C15B	32.2 (2)
O4A—S1A—C14A—C15A	158.6 (2)	O4B—S1B—C14B—C15B	162.2 (2)
N1A—S1A—C14A—C15A	−86.5 (3)	N1B—S1B—C14B—C15B	−83.0 (2)
O3A—S1A—C14A—C19A	−152.9 (2)	O3B—S1B—C14B—C19B	−150.0 (2)
O4A—S1A—C14A—C19A	−23.2 (3)	O4B—S1B—C14B—C19B	−20.0 (2)
N1A—S1A—C14A—C19A	91.7 (2)	N1B—S1B—C14B—C19B	94.8 (2)
C19A—C14A—C15A—C16A	−1.5 (5)	C19B—C14B—C15B—C16B	−0.7 (4)
S1A—C14A—C15A—C16A	176.8 (3)	S1B—C14B—C15B—C16B	177.1 (2)
C14A—C15A—C16A—C17A	−0.3 (6)	C14B—C15B—C16B—C17B	0.7 (5)
C15A—C16A—C17A—C18A	1.9 (5)	C15B—C16B—C17B—C18B	0.3 (5)
C15A—C16A—C17A—C20A	−177.9 (4)	C15B—C16B—C17B—C20B	−179.4 (3)
C16A—C17A—C18A—C19A	−1.7 (5)	C16B—C17B—C18B—C19B	−1.2 (4)
C20A—C17A—C18A—C19A	178.1 (3)	C20B—C17B—C18B—C19B	178.4 (3)
C17A—C18A—C19A—C14A	0.0 (4)	C17B—C18B—C19B—C14B	1.2 (4)
C15A—C14A—C19A—C18A	1.7 (4)	C15B—C14B—C19B—C18B	−0.2 (4)
S1A—C14A—C19A—C18A	−176.6 (2)	S1B—C14B—C19B—C18B	−178.0 (2)

### Hydrogen-bond geometry (Å, °)

**Table:**

*D*—H···*A*	*D*—H	H···*A*	*D*···*A*	*D*—H···*A*
C4B—H4B2···O4A^i^	0.97	2.53	3.277 (3)	134
C13A—H13B···O5B^ii^	0.96	2.51	3.256 (5)	134
C13A—H13C···O4B^iii^	0.96	2.58	3.229 (4)	125
C18A—H18A···O3A^iv^	0.93	2.54	3.409 (4)	156
C18B—H18B···O3B^v^	0.93	2.56	3.403 (3)	151
C5A—H5A2···O4A	0.97	2.41	2.825 (3)	105
C6A—H6A···O3A	0.98	2.40	2.908 (3)	112
C6A—H6A···O5A	0.98	2.45	2.820 (3)	102
C6B—H6B···O3B	0.98	2.44	2.939 (3)	111
C6B—H6B···O5B	0.98	2.34	2.808 (3)	109
C5B—H5B2···O4B	0.97	2.47	2.818 (3)	101
C19A—H19A···O4A	0.93	2.56	2.917 (4)	104
C19B—H19B···O4B	0.93	2.54	2.909 (3)	104

Symmetry codes: (i) −*x*+1, *y*+1/2, −*z*+1/2; (ii) −*x*+1/2, −*y*+1, *z*−1/2; (iii) *x*+1/2, −*y*+3/2, −*z*+1; (iv) *x*−1, *y*, *z*; (v) *x*+1, *y*, *z*.
